# Takayasu Arteritis Complicated by Superior Mesenteric Artery Stenosis and Bilateral Renal Arteritis: A Clinical Case Report

**DOI:** 10.7759/cureus.72477

**Published:** 2024-10-27

**Authors:** Mallikarjuna S Oruganti, Mohammed Haroon Ahmed, Ayman Nadeem, Manasi Narreddy, Sumana Simarla, Katta Manideep, Krishna S Athmakuri, Asma Farheen Sheik, Saieesh Bairam, Sameena Tabassum

**Affiliations:** 1 Internal Medicine, Osmania Medical College, Hyderabad, IND; 2 Radiodiagnosis, Gandhi Medical College, Hyderabad, IND; 3 General Surgery, Osmania Medical College, Hyderabad, IND; 4 Pediatrics, Osmania Medical College, Hyderabad, India; 5 Radiology, Osmania Medical College, Hyderabad, IND; 6 Pediatrics, All India Institute of Medical Sciences, Mangalagiri, Mangalagiri, IND

**Keywords:** aortic wall thickening, ct aortogram, large vessel vasculitis, narrowing of aorta, stenosed renal artery, superior mesenteric artery (sma), takayasu arteritis (tak), upper extremity claudication

## Abstract

Takayasu arteritis (TA), a rare large-vessel vasculitis, primarily affects women of childbearing age, causing granulomatous inflammation in the aorta and its major branches. This inflammation can lead to stenosis, aneurysms, or occlusion, with the abdominal aorta, subclavian, and brachial arteries commonly involved. We present the case of a 26-year-old female with TA with a rare involvement of the superior mesenteric artery (SMA). The patient presented with progressive shortness of breath and exertional pain in the left upper limb, suggesting claudication. Echocardiography showed an ejection fraction of 45% and right ventricular systolic pressure of 65 mmHg. CT angiography (CTA) revealed diffuse involvement of the abdominal aorta with non-opacification of SMA, and other findings were consistent with TA type 5. The patient was successfully treated with methylprednisolone to induce remission, along with guideline-directed medical therapy for heart failure.

## Introduction

Takayasu arteritis (TA) is a rare, chronic inflammatory vasculitis primarily affecting large arteries, especially the aorta and its main branches. First identified in 1908 by Japanese ophthalmologist Dr. Takayasu, this disease involves complex immune responses, vascular changes, and genetic factors. Inflammation begins near the vasa vasorum and at the media-adventitia junction, causing panarteritis. This results in arterial wall thickening, stenosis, blockages, and aneurysm formation. TA progresses through three stages: the "pre-pulseless" phase (Stage I), the "pulseless" phase (Stage II), and the "fibrotic" phase (Stage III). The symptoms vary and include systemic signs and vascular issues like weak or absent pulses, blood pressure differences between arms, vascular bruits, and localized tenderness [1].

## Case presentation

A 26-year-old female was admitted to our hospital with complaints of dyspnea and pain in the left upper limb for the past three months. The patient initially had mild dyspnea on exertion, which had gradually progressed. Over the past week, her respiratory symptoms had worsened significantly, leading to orthopnea and paroxysmal nocturnal dyspnea. Additionally, she had experienced episodes of giddiness, generalized weakness, and mild intermittent fever (99^o ^F) over the last month. Over the past 24 hours, she had been unable to carry out her daily activities due to severe claudication pain in her left upper limb, as it particularly worsened with activity and subsided on rest.

On physical examination, a comprehensive head-to-toe assessment revealed pallor. The patient was hypertensive, with a blood pressure of 150/90 mmHg measured in the right upper limb. In contrast, the left upper limb exhibited a weak pulse, and its blood pressure was unrecordable. Blood pressure in the lower limbs was 140/130 mmHg on the right and 160/130 mmHg on the left. The pulse rates were 114 beats per minute (bpm) in the right upper limb, 126 bpm in the right lower limb, and 121 bpm in the left lower limb, with normal characteristics in terms of volume, character, and rhythm. Despite experiencing dyspnea, the patient maintained an oxygen saturation (SpO_2_) of 96% on room air. Auscultation of the lung fields and precordium did not reveal any murmurs or crackles.

All the laboratory tests returned normal except for the parameters mentioned in Table 1.

**Table 1:** Key positive laboratory findings

Investigation	Patient value	Normal range
Hemoglobin	6.6 g/dL	12.0-16.0 g/dL
Red blood cell count	2.7 million/mm^3^	3.5-5.5 million/mm^3^
Total leukocyte count	4200/mm^3^	4500-11000/mm^3^
Platelet count	274,000/mm^3^	150,000-400,000/mm^3^
Erythrocyte sedimentation rate (ESR)	38 mm/hr	0-20 mm/hr
C-reactive protein (CRP)	14 mg/l	8-10 mg/l
Blood urea nitrogen (BUN)	91 mg/dL	7-18 mg/dL
Creatinine	3.15 mg/dL	0.6-1.2 mg/dL
Aspartate aminotransferase (AST)	137 U/L	12-38 U/L
Alanine aminotransferase (ALT)	79 U/L	10-40 U/L
Alkaline phosphatase (ALP)	169 U/L	25-100 U/L

Imaging findings and impressions

An echocardiogram demonstrated severe pulmonary artery hypertension, with right ventricular systolic pressure elevated to 65 mmHg, and dilatation of the right atrium and ventricle. Additionally, there was severe tricuspid regurgitation, moderate aortic regurgitation, and mild mitral regurgitation, while the left ventricle showed mild dysfunction with an ejection fraction of 45%.

**Figure 1:**
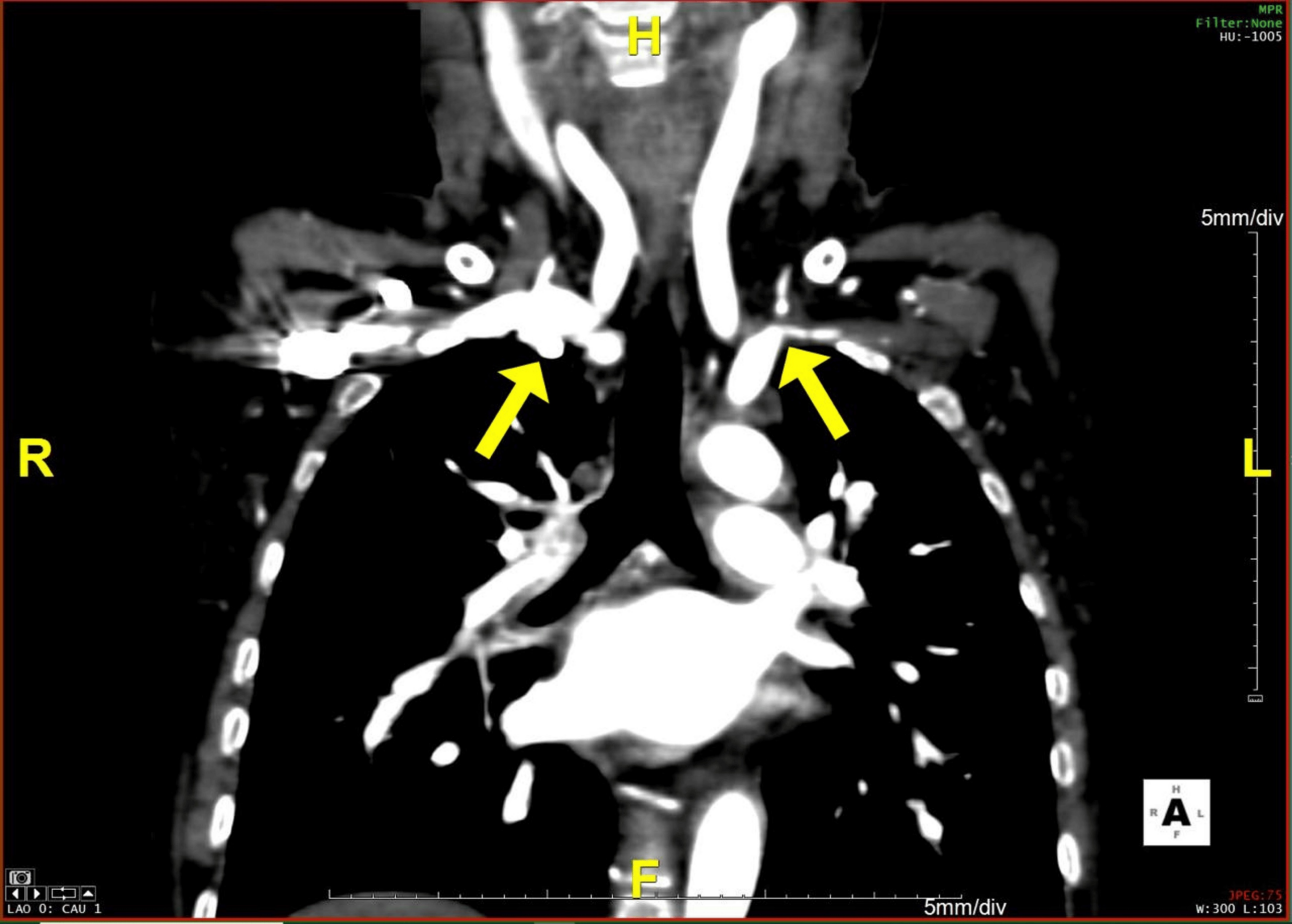
A coronal section of the CT angiogram comparing both subclavian arteries The image shows stenosis in the left subclavian artery CT: computed tomography

**Figure 2:**
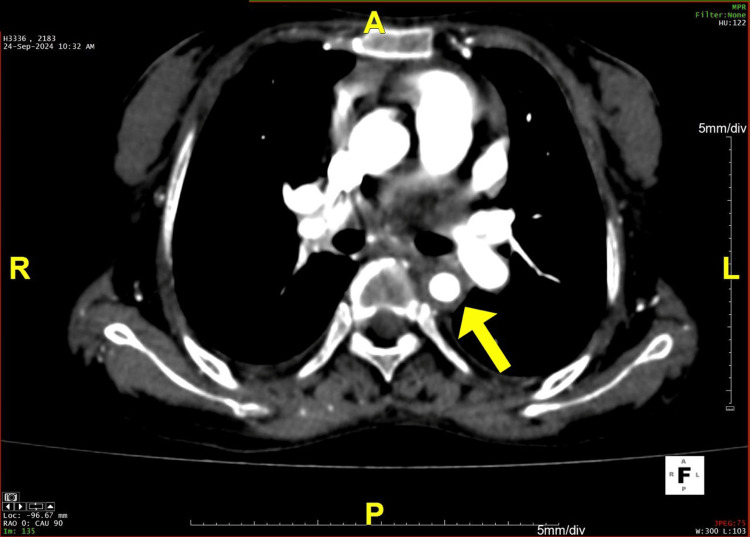
An axial section of the CT angiogram shows narrowing of the descending thoracic aorta CT: computed tomography

**Figure 3:**
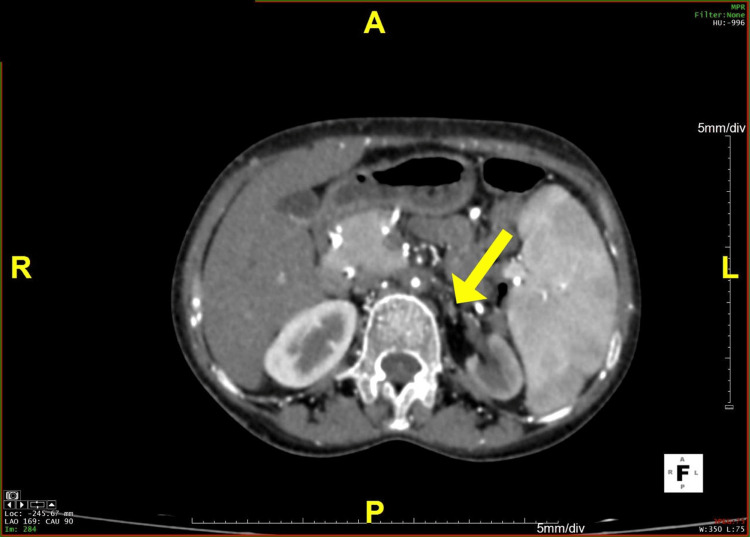
An axial section of the CT angiogram revealing severe stenosis with non-opacification of the left renal artery CT: computed tomography

**Figure 4:**
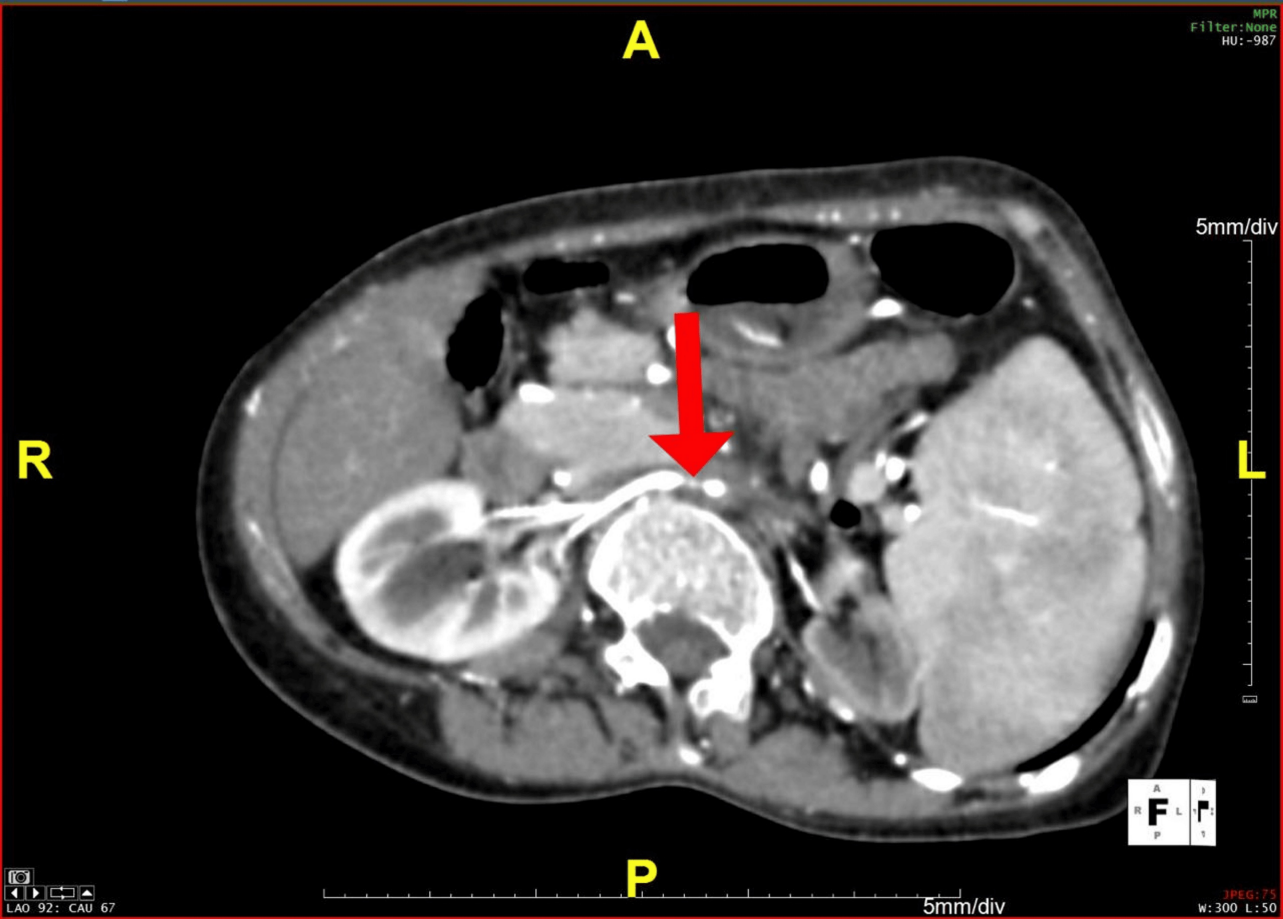
An axial section of the CT angiogram demonstrating stenosis of right renal artery at its origin CT: computed tomography

**Figure 5:**
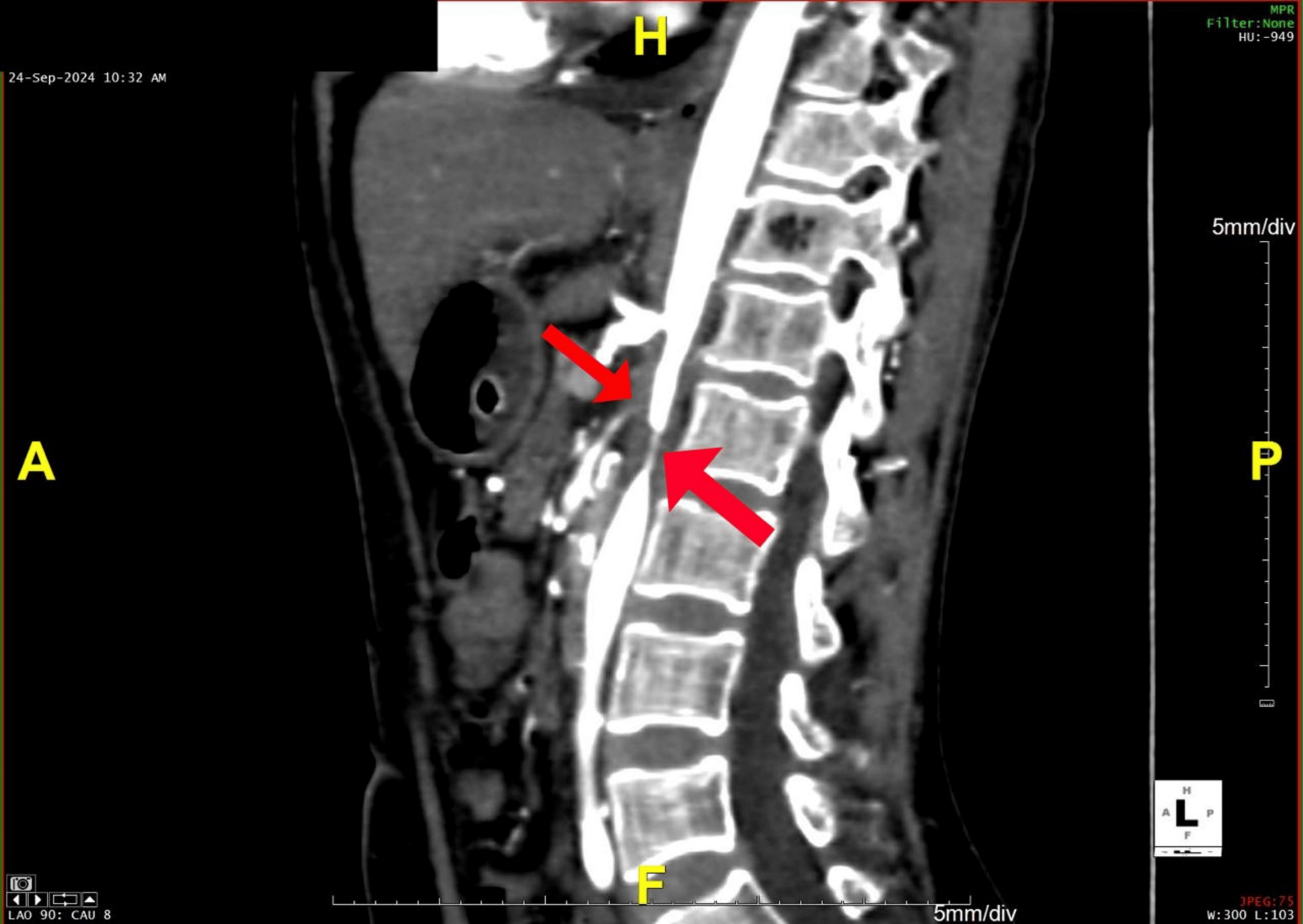
A sagittal section of the CT angiogram reveals narrowing of the abdominal aorta and superior mesenteric artery CT: computed tomography

**Figure 6:**
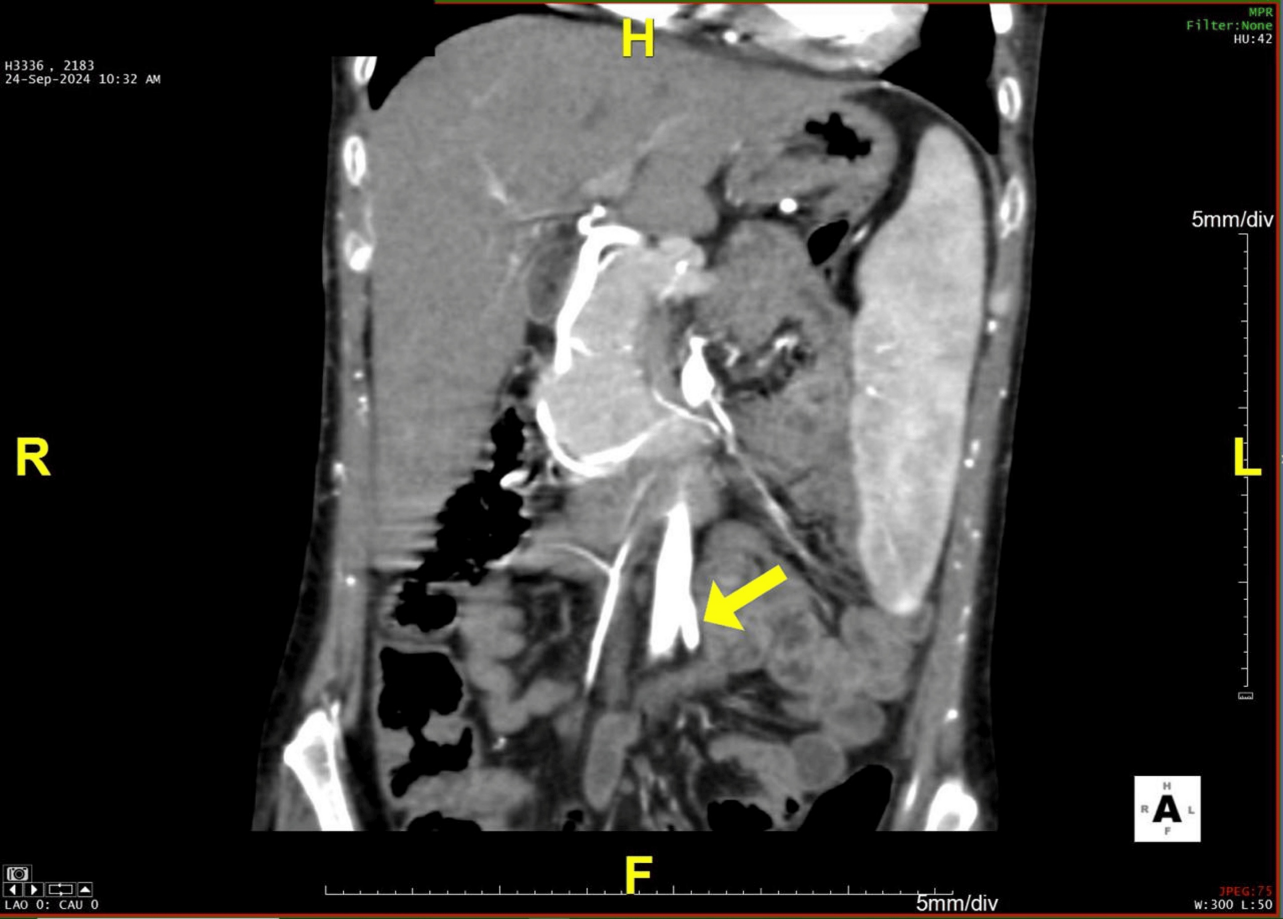
A coronal section of the CT angiogram reveals sparing of the inferior mesenteric artery CT: computed tomography

Doppler ultrasound evaluation of the left upper limb claudication revealed diffuse thickening of the vessel walls in the left subclavian artery (SCA), axillary artery (AA), and brachiocephalic artery (BA), all showing biphasic waveforms. In contrast, the radial artery (RA) and ulnar artery (UA) displayed monophasic waveforms. A CT aortic angiogram was then performed using 100 cc of non-ionic contrast medium with a bolus tracking technique to assess for additional vascular involvement. The findings (Figures 1-6) revealed diffuse long-segment stenosis (66-77% luminal narrowing) of the abdominal aorta over a length of 5.4 cm, extending from the celiac trunk to the renal arteries. Additionally, there was significant stenosis of the left SCA distal to the origin of the vertebral artery (greater than 70% luminal narrowing), severe narrowing of the ostium of the right renal artery with noted collaterals from the celiac artery (CA), and non-opacification of the proximal segment of the superior mesenteric artery (SMA), with reformation of the distal SMA supplied by collateral vessels from the inferior mesenteric artery (IMA).

Treatment and follow-up

The patient received treatment for heart failure with enalapril (2.5 mg/day), spironolactone (25 mg/day), metoprolol 25 mg/day, and furosemide (80 mg/day, divided into 40 mg and two 20 mg doses administered every eight hours). Heparin (5000 units IV every six hours) was initiated to prevent thromboembolism. A high-dose oral methylprednisolone regimen (1 mg/kg/day) was started, leading to the normalization of inflammatory markers within four weeks and the improvement of claudication symptoms. The methylprednisolone dosage was then gradually tapered to a maintenance dose of 18 mg/day. However, the patient was subsequently lost to follow-up, and hence, post-treatment echocardiogram and CT angiogram (CTA) studies were not conducted.

## Discussion

TA is a large-vessel granulomatous vasculitis that is idiopathic in most cases [2]. It often occurs in the brachial, subclavian, and renal arteries, but rarely in the celiac or superior mesenteric arteries [3]. Chronic inflammation results in fibrosis, intimal proliferation, neovascularisation, and degeneration of elastic fibers, resulting in stenosis and aneurysm of involved vessels. There is also evidence of demographic associations of the pattern of vessels involved, with aortic arch involvement most common in the Japanese population, and abdominal aorta and renal artery involvement in Indians [4]. Among the three main aortic branches, the left common carotid and left subclavian are commonly affected in most cases [5]. Our patient presented with left subclavian stenosis alone.

TA initially presents with nonspecific prodromal features such as malaise, fever, anorexia, weight loss, elevated ESR and CRP, and vascular pain. At later stages, it may present characteristically with occlusive symptoms such as limb claudication, ‘pulselessness’, vascular bruit, differences in systemic pressures between both limbs, renal artery stenosis resulting in renovascular hypertension, bowel ischemia, transient ischemic attack, stroke, and myocardial infarction [4]. Visceral disease can manifest as acute abdominal pain or acute exacerbations of chronic abdominal pain, which often leads to misdiagnosis [3].

CTA has replaced conventional angiography as the gold standard and is used for initial staging, enabling visualization of wall thickening and luminal narrowing. The findings are used to classify the disease, allowing the comparison of patient characteristics, assessing the extent of the disease based on the vessels involved, and helping develop a management plan. Diagnosis is based on the 1990 American College of Rheumatology criteria, a combination of clinical and angiographic findings, with our case scoring a total of 6 (the maximum). A minimum of 3 out of 6 is required to establish a diagnosis. The Ishikawa criteria may also be used. Further subclassification is done using the Numano classification, or the 1994 New angiographic classification of Takayasu arteritis, based on which our case can be determined to be type 5, a combination of types 2b and 4, with involvement of the abdominal aorta, renal arteries, and the left subclavian [6]. 

Of note, 11% to 68% of patients have stenotic lesions in the visceral arteries, and SMA involvement (11-21.4%) is rare compared to the abdominal aorta and renal arteries [7]. The involvement of the SMA and CA and sparing of the IMA, allowing it to act as collateral, has been reported, and the presence of collaterals also enables asymptomatic disease. The IMA and SMA being involved simultaneously is rare and has only been reported twice, by Nawghare et al. and Nogueira et al. [2,8]. When collaterals are affected, the development of ischemic manifestations begins, and, at presentation, the management can be difficult. TA is often associated with anemia, particularly during the active inflammatory stage, when IL-6 induces the production of hepcidin, an acute-phase reactant, leading to the inhibition of iron absorption, manifesting as anemia. Pulmonary vessel involvement leads to chronic hypoxia and stimulates erythropoiesis, and is usually not observed in patients presenting with anemia, suggesting that our patient presented in the active stage, based on her elevated ESR and CRP levels, as well as CTA findings [9]. 

Patients with TA are at a high risk of cardiovascular events especially when associated with HLA-Bw52 [10]. Involvement of the ascending aorta leads to dilatation of the aortic root with or without valvular involvement, resulting in aortic regurgitation. However, heart failure itself in TA can be due to multiple complications - aortic regurgitation, renovascular hypertension, coronary artery involvement, and accelerated atherosclerosis. In our case, severe pulmonary hypertension (right ventricular systolic pressure of 65 mmHg) was likely due to acute aortic regurgitation (no LA dilation) resulting in left ventricular dysfunction with mitral regurgitation, given that the pulmonary artery was not involved. Severe pulmonary hypertension progressed to right ventricular enlargement, tricuspid regurgitation, and right atrial dilation. This explains our patient’s complaints of dyspnea, orthopnea, and paroxysmal nocturnal dyspnea. The risk of aortic regurgitation and pulmonary hypertension is of particular concern in patients with active disease [11]. Given that our patient had an ejection fraction of 45%, she was diagnosed with heart failure with a mildly reduced ejection fraction (HFmrEF). The management of hypertension and heart failure secondary to regurgitation and high-grade stenosis of the aorta and subclavian vessels does not differ from that of a patient without TA [12].

Medical management targets vasculitis, and the European League Against Rheumatism (EULAR) in 2018 recommended the induction of early remission with steroids and immunosuppressants such as azathioprine, methotrexate, mycophenolate mofetil, cyclophosphamide, and leflunomide as adjuncts [13]. Prednisolone is the recommended first-line agent, and immunosuppressants, as well as biological actives such as Infliximab, tocilizumab, and etanercept, are often required to prevent the adverse effects of long-term steroid use and mitigate the risk of relapse. Statins have also been reported to reduce relapse rates in TA patients [14]. This is crucial as TA has been known to progress even when clinically inactive, implying that medical therapy may not be as effective at arresting disease as previously thought, and this has led to surgical interventions being designated as primary treatment options [15]. Percutaneous transluminal angioplasty is an effective short-term strategy for symptomatic relief in patients with other conditions taking precedence, and in open surgery, reconstruction is performed with polytetrafluoroethylene or dacron for major aortic reconstruction, and saphenous vein grafts for extremities and isolated renal or mesenteric involvement [16]. 

Stable remission allows for surgical intervention, with endovascular methods like balloon dilatation, stenting, and bypass becoming popular due to their minimally invasive approach. Surgery is often successful, particularly in young patients, leading to symptom improvement. The choice of invasive therapy depends on lesion severity and response to medical treatment [3].

## Conclusions

TA is a large-vessel granulomatous vasculitis prevalent among young females and has a varied presentation. Doppler ultrasound of vessels along with a CT aortic angiogram can detect the rare involvement of SMA and bilateral renal artery stenosis. A thorough knowledge of the varied presentations, investigations to assess the multiorgan involvement, and multidisciplinary management involving physicians, rheumatologists, and cardiologists are needed for the initial stabilization of the patient and may help in the long-term management of the condition. Corticosteroids were the mainstay of treatment in the management of TA in our patient, which induced remission of symptoms with a high-dose oral methylprednisolone regimen (1 mg/kg/day), leading to the normalization of inflammatory markers within four weeks and improvement in claudication symptoms. However, the patient was lost to follow-up, and post-treatment echocardiogram and CT angiogram assessments could not be performed.

## References

[1] Bhandari S, Butt SR, Ishfaq A (2023). Pathophysiology, diagnosis, and management of Takayasu arteritis: a review of current advances. Cureus.

[2] Nawghare P, Thanage R, Jain S, Chandnani S, Rathi PM (2022). Takayasu arteritis presenting as intestinal angina: unusual presentation of a rare disease. Cureus.

[3] Zheng Y, Wu S, Tong W, Chen Z, Cheng M (2018). Superior mesenteric artery and celiac artery stenosis caused by Takayasu arteritis: a case report and review of the literatures. J Am Coll Cardiol.

[4] Chaudhry MA, Latif F (2013). Takayasu's arteritis and its role in causing renal artery stenosis. Am J Med Sci.

[5] Chung JW, Kim HC, Choi YH, Kim SJ, Lee W, Park JH (2007). Patterns of aortic involvement in Takayasu arteritis and its clinical implications: evaluation with spiral computed tomography angiography. J Vasc Surg.

[6] Setty HSN, Vijaykumar JR, Nagesh CM, Patil SS, Jadav S, Raghu T, Manjunath CN (2017). Takayasu's arteritis - a comprehensive review. J Rare Dis Res Treat.

[7] Yamashita S, Nagao K, Doi T, Yokoyama S, Yamashita A, Fukahara K, Yoshimura N (2022). Takayasu arteritis complicated by ischemic colitis: a case report. Ann Vasc Dis.

[8] Nogueira Rde C, Oliveira EF, Conforto AB, Bor-Seng-Shu E, Puglia P, Lucato LT, Marchiori PE (2012). Takayasu's arteritis and cerebral venous thrombosis: comorbidity or coincidence?. Arq Neuropsiquiatr.

[9] Zhang Y, Zhang D, Qu Y (2019). Anemia in patients with Takayasu arteritis: prevalence, clinical features, and treatment. J Geriatr Cardiol.

[10] Serra R, Butrico L, Fugetto F (2016). Updates in pathophysiology, diagnosis and management of Takayasu arteritis. Ann Vasc Surg.

[11] Lee GY, Jang SY, Ko SM (2012). Cardiovascular manifestations of Takayasu arteritis and their relationship to the disease activity: analysis of 204 Korean patients at a single center. Int J Cardiol.

[12] Suryono S, Wulandari P, Ariyanti D, Maulana AS, Sembodo RH, Junior NW, Saputra AD (2022). Takayasu arteritis with congestive heart failure in 26-year-old male: a case report. Egypt Heart J.

[13] Hellmich B, Agueda A, Monti S (2020). 2018 Update of the EULAR recommendations for the management of large vessel vasculitis. Ann Rheum Dis.

[14] Kwon OC, Oh JS, Park MC, Hong S, Lee CK, Yoo B, Kim YG (2019). Statins reduce relapse rate in Takayasu arteritis. Int J Cardiol.

[15] Aeschlimann FA, Eng SW, Sheikh S (2017). Childhood Takayasu arteritis: disease course and response to therapy. Arthritis Res Ther.

[16] Mason JC (2018). Surgical intervention and its role in Takayasu arteritis. Best Pract Res Clin Rheumatol.

